# Development and Psychometric Properties of Social Exclusion Questionnaire for Iranian Divorced Women

**Published:** 2017-05

**Authors:** Fatemeh ZAREI, Mahnaz SOLHI, Effat MERGHATI-KHOEI, Mohammad Hossein TAGHDISI, Davoud SHOJAEIZADEH, Ann Rosemary TAKET, Razieh MASOOMI, Saharnaz NEDJAT

**Affiliations:** 1. Dept. of Health Education and Health Promotion, School of Public Health, Zanjan University of Medical Sciences, Zanjan, Iran; 2. Dept. of Health Education and Health Promotion, School of Public Health, Iran University of Medical Sciences, Tehran, Iran; 3. Iranian National Center of Addiction Studies (INCAS), Sexual and Family Health Division, Tehran University of Medical Sciences, Tehran, Iran; 4. Center for Health Through Action on Social Exclusion, School of Health and Social Development, Deakin University, Melbourne, Australia; 5. Dept. of Reproductive Health, School of Nursing and Midwifery, Tehran University of Medical Sciences, Tehran, Iran; 6. Dept. of Epidemiology and Biostatistics, School of Public Health, Tehran University of Medical Sciences, Tehran, Iran

**Keywords:** Social exclusion, Divorce, Women, Validity, Iran

## Abstract

**Background:**

Divorce, especially in women, could be assessed from socio-cultural perspective as well as psychological viewpoint. This assessment requires cultural adopted as well as valid and reliable questionnaire. This study aimed to develop and assess the psychometric properties of a questionnaire in order to address social consequences in Iranian divorced women.

**Methods:**

This was an exploratory mixed method study conducted during 2012 to 2014. According to the grounded theory approach in the first phase, social exclusion was extracted as a core of understanding process in participants. Based on, 47 preliminary generated items reliability and validity were assessed. In the second phase, the divorced women were recruited from a safe community center in Tehran through convenience sampling.

**Results:**

Exploratory factor analysis conducted on the questionnaires of 150 divorced women with mean age 41.76±8.49 yr, in that, indicated five dimensions, discriminative marital status, economic dependence on marital status, exclusionary marital status, and traumatic marital status health risks and, frightening marital status that jointly accounted for the 64% of the variance observed. An expert panel approved the face and content validity of the developed tool. The Cronbach’s alpha coefficient and the Intra-class Correlation Coefficient were found to be 0.70 and 0.85, respectively.

**Conclusion:**

The present study provided a valid and reliable measure as Social Exclusion Questionnaire in Iranian divorced women (SEQ-IDW) to address social post-divorce consequences, which might help to improve women’s social health.

## Introduction

Divorce is an unforeseen change in the family composition and has long-term outcomes for the individual and society (1). Divorce means the abolishment of legal duties and responsibilities of marriage and separation of partners. Although there are identical definitions for divorce in literal and legal terms, the emergence and understanding of this phenomenon differ in the cultural context of each society, due to its complex nature (2).

There exists a vast literature on the increase in the aggregate divorce rate. Thus, a crude divorce ratio in the USA increased from 2.2 in 1960 to 5.2 in 1980 and then, after the early 1980s, it decreased to 3.5 in 2008. However, it is still one of the most probable events in the life cycle events (3). Divorce has an incremental trend in Iran as well. The percentage of households that are female-headed has increased from 7% in 1986 to 12% in 2011; this ratio shows increased divorce rate and number of single-headed households (Iran Census Bureau 2011). According to the latest census in 2011, the number of divorced women in Iran was 4.1% and almost twice as much as men in the over 10-year-old population. Currently, Iran ranks fourth in terms of divorce in the world so that, in 2013, one out of every five marriages in Iran and one out of 3 in Tehran ended in divorce (4).

Social exclusion is one of the social factors affecting health, which affects achieving equal life opportunities (5). Moreover, separation of parents was considered as one of the processes leading to social exclusion and also refers to loss of equal opportunities, social stigma, discrimination, unemployment, marginalization, and loneliness as the outcomes of depriving processes (5). Social exclusion in Japan was determined in terms of the emergence of different communal deprivation dimensions such as financial poverty, denial of public services, lack of social interaction, inappropriate housing, lack of activities, and loneliness (6). Furthermore, some of the characteristics of social exclusion were introduced as inability to participate in socio-cultural, economic, and political activities, inequality in accessing commodity and resources, income, and services, unemployment, weakness in skills, low income, inappropriate housing, increased probability of crime, health poverty, transportation limitation, as well as inability in social interactions with family, friends, and society (7).

A study on divorce among Iranian women confirm the divorce as socially excluding process and identifies inequality in accessing occupational opportunities, having social ties, losing family and social support, social and family ostracism, and damage to health as the most important outcomes of this depriving process (8). Thus, according to the lived experiences of participants in the qualitative part of this study, the deprivation process of divorce goes beyond political and economic components and to socio-cultural factors (9). Divorce is a more complicated issue than the separation of two people and goes beyond individualistic and psychological outcomes (10). Therefore, detecting and understanding divorce and its resulting consequences need a deeper and deliberate view derived from lived perceptions and experiences.

Self-report questionnaires could be an appropriate method to evaluate the social exclusion of divorced women. Since this concept is subjective as well as self-judgmental. There are two types of questionnaires used for assessing the sensitive concepts: general and specific questionnaires. Specific tools could be more useful than general types because they show higher sensitivity and specificity (14). Since a tool is needed for every intervention and evaluation, this study attempted to design a tool for measuring changes in social exclusion following divorce.

There are two approaches to design and determine the validity of a questionnaire. In the first one, the available tools and resources are used, translated from one language into another. In the second approach, a new tool is designed based on the facts and experiences rooted in the culture of each society. In the second approach, cultural considerations are the main basis for the questionnaire formulation (11). The assumption of the present study was that the available resources for designing reliable tools are not sufficient and suitable and in order to explain the social exclusion following divorce among Iranian women; inductive approach of grounded theory was conducted to identify lived experiences of the participants without applying preconceived dimensions or existing theoretical viewpoints.

This study is a mixed-method research, which designed and validated a social exclusion questionnaire derived.

## Materials and Methods

The present study was an exploratory mixed method study. Indeed, it has been designed in two phases. First, a qualitative method was applied to generate items and develop the questionnaire. Second, psychometric properties of the questionnaire were measured.

### Phase one: Item generation and questionnaire development

In this qualitative inquiry, we applied grounded theory approach. Data were collected using focus group discussions and individual interviews between Jun and Mar 2012.

### Participants and data collection

We recruited a group of divorced women (n=26) aged 25–55, who volunteered to participate in the study. Women were selected purposefully from District 21 of Tehran Municipality. Participants were informed of the objectives of the study and then interviews were conducted. Participants had at least a high school educational level. Most were living in a rental house alone. A few of them were living with their families at the time of the interviews.

We developed focus group discussions as the primary method of data collection. The sessions were facilitated by defining divorce, and using a semi-structured inventory that began with the open-ended questions: ‘How is life as a divorced Iranian woman’. Then, based on the responses obtained from the participants, subsequent questions built upon the discussion. However, the participants with different levels of experiences challenged each other’s viewpoint in learning and understanding divorce. We documented our analytic ideas by writing memos. The focus group discussions enabled the researchers to identify the potential informants for individual interviews. Those women who had specific experience, for instance, temporary marriage (Siqa) or sexual activity after divorce were explored during FGDs and invited for individual interviews (4 out of 26 participants). Although women who had experience of temporary marriage and sexual activity did not speak openly in the discussion, we explored them when they spoke about their beliefs. For example, having experience about temporary marriage is an available way to respond a woman’s needs after divorce (9). Furthermore, in order to have access to other divorced women with experience of temporary marriage, snowball sampling was used, and additional divorced women were identified for individual interviews. In-depth, individual interviews delivered a situation for us to speak about their beliefs and experiences. Each FGD lasted for 45 to 70 min, and the second appointment was made if needed. In total, 10 focus group discussions were held, and four participants were interviewed individually. Data saturation was achieved after seven focus group discussions and three individual interviews. Sampling was continued with maximum variation to yield greater transferability of data and saturation (12). To achieve maximum variation, informants were selected from different age groups, distinctive socioeconomic backgrounds, having various types of experiences in divorce, and being as high and low level in religiosity.

### Data analysis

We employed grounded theory as an approach that enables researchers to explore a person’s life experience in their real contexts and societal interactions (14). Analysis of the transcripts was guided by constant comparative analysis (13, 14). Each transcript was read and reexamined line by line to identify free codes. Coding was used here as a means of recognizing and abstracting events, actions, and meanings. During the analysis, the researcher reflected on connotations and perceptions using a memo created after each interview. Another round of discussion among the researchers led to and revised version of the category list. In total, 1 core, 5 key themes, and 12 sub-themes emerged as important factors relating to social exclusion in divorced women. The framework has been revealed in Table 1.

**Table 1:** Core, themes, and sub-themes explored by the focus groups and individual interviews (Phase 1)

**Core**	**Theme**	**Sub-Theme**
	Social Unreliable	Lake of Family Support
		Lake of social support
		Lake of Self-efficacy
		Being divorced as an untrustworthy adjective
Social Rejection	Negative Social states of mind	Social Stigmatizing
		Social discrimination for divorced woman suitability for Temporary marriage
		Health risk behavior after divorce
	Social Alienation	Social identity treated
		Sexual identity treated
	Suspended social communication	Communicating with others
		Staying away from others

The trustworthiness of the discoveries was recognized as follows. After coding the data, the transcripts and codes were examined, five of the participants and colleagues who conducted member checking.

The researchers converted about similarities and differences in their coding and compared classifications to progress a preliminary category list. The list of categories was compared with the original copies, probing for data that supported or rejected the preliminary categories (15).

From women under study perspective, five themes developed; 1) Unreliability, 2) Social states of mind 3) rejection and Isolation 4) Alienation, and 5) Suspension. we found that in Iran divorced women are sexually judged and considered dangerous sexual objects, causing promiscuity (16).

Divorced women attempt to avoid this judgment by looking for exclusion. Most of them purposely stop socialization after separation. They occasionally go to parties where women accumulate with their husbands. Then again, wedded women disengage their associations with their separated companions to protect their spouses.

A Persian figure of speech says: “Woman comes to bride house with white gown and leaves the marriage with white coffin” which implies marriage is long lasting. A divorcee woman is one who digresses from this script rejected by the individuals from the group.

Social isolation and social exclusion came about because of the evasion of stereotypic cooperation’s and were frequently a result of challenges with camouflage of sexual needs because of stigma. Women talked about a scope of undesirable encounters emerging in light of their needs in post-divorce life (Table 1). According to the findings extracted from phase one, the questionnaire entitled Social Exclusion Questionnaire in Iranian Divorced Women (SEQ-IDW). Finally, pool containing 47 statements was generated applied for psychometric properties.

### Phase two: Psychometric properties of the SEQ-IDW

The pre-final draft of the SEQ-IDW contained 47 items, and each item is rated on a five-point response questionnaire (completely agree to completely disagree).

### Design and Data Collection

This was a cross-sectional study with convenience sampling including 150 divorced women were recruited through a safe community center in West Tehran, Iran, established in 2010. Women who mainly resident in District 21 was provided an amount of money on a regular basis by Social Deputy of Women’s Affaire in Tehran, Iran from Apr to July 2013. Individuals were eligible for recruitment if they were at least 25 yr old, with one divorce experience, and minimum literacy skills. The participants who did not like answering the questionnaires were excluded. The sample size was estimated based on the final number of items in the questionnaire multiplying by 5 as recommended. The participants were informed that take over the study was voluntary; their confidentiality would be preserved, and none of the participants would be recognized in any publications resulting from this study. Informed consent document was obtained from all participants.

### Statistical analysis

Psychometric properties of the questionnaire were assessed by several statistical tests as follows:

### Validity

We assessed content, face, and construct validity of the SEQ-IDW as follows:

### Content validity

Qualitative and quantitative content validity was applied. An expert panel consisting of a team of investigators specialized in health promotion and psychometrics assessed the content validity of the questionnaire. In the qualitative phase, they appraised phrasing, grammar, item allocation, and scaling of the questionnaire (17).

In the quantitative phase, both the content validity index (CVI) and the content validity ratio (CVR), were considered. Clarity, simplicity, and relevance of each item are assessed by CVI evaluation (15, 18). In order to calculate the CVI, we used a Likert-type, ordinal questionnaire with four probable responses. The answers contain a rating from 1 =no relevant, no simple and no clear to 4=completely relevant, completely simple and completely clear. According to the received expert panel, each item on a 3 or 4 was considered as the part of items in CVI (19). The essentiality of items was tested by calculating the CVR. For calculating the CVR, the experts rated each item as essential, useful but not essential, or not essential (20).

### Face validity

qualitative and quantitative methods were applied to assess face validity. In the qualitative phase, 20 divorced women were asked to assess the questionnaire and indicate if they find difficulty or ambiguity in responding to the questionnaire.

In the quantitative phase, the item impact (frequency × importance) was calculated to specify the percentage of divorced women who identified the item as important or quite important. Items were considered suitable if they had an impact score equal to or more than 1.5 (which matches to a mean frequency of 50% and a mean importance of 3 on the 5-point Likert questionnaire)

### Construct validity

Exploratory factor analysis (EFA) was applied to determine the real but not immediately obvious constructs of the questionnaire. A principal component analysis (PCA) with varimax rotation was applied and the factor loading equal to or greater than 0.3 was considered acceptable (17).

### Reliability

To measure the internal consistency of the questionnaire, the Cronbach’s alpha coefficient was calculated. Values equal to or upper than 0.70 were considered acceptable (21) Furthermore, so as to estimate the questionnaire’s stability, Test-retest reliability was conducted frothy participants completed the questionnaire twice with 10-d intervals. The intraclass correlation coefficient (ICC) values of 0.40 or upper were considered acceptable (rμ 0.81–1.0 as excellent, 0.61–0.80 very good, 0.41–0.60 good, 0.21–0.40 fair, and 0.0–0.20 poor) (22, 23).

### Ethics

Approval to conduct the study was granted by the Ethics Committee of the Tehran University of Medical Sciences, Tehran, Iran.

## Results

Totally, 150 divorced women completed the SEQ-IDW. The mean age of participants was 41.76±8.49 yr. The demographic characteristics of study sample are shown in Table 2. The total score for each participant was calculated by summing the items.

**Table 2:** Characteristics of the study sample (phase 2, n=150)

		**Number**	**Percent**
**Reason for divorce**			
	Infidelity	31	20.7
	Addiction of couple	67	44.7
	Psychological disorder	10	6.7
	Others	38	25.4
**Education**			
	Illiterate	7	4.7
	Primary school	15	10.0
	Intermediate school	18	12.0
	High school	91	60.6
	University graduate	19	12.7

### Validity

#### Content validity

In the quantitative content validity phase, items with CVR and CVI less than 0.62 and 0.80, respectively, were omitted (17 items). In the qualitative phase, other criteria such as grammar, wording, and item allocation were edited according to the experts’ opinions. For instance, the sentence “Divorce will bring social label” changed to “Divorce carries stigma”, in addition, “Being divorced is barrier to equal employment opportunity” changed to “Being divorce is a barrier to achieving equal job opportunity”.

#### Face validity

Impact score was calculated to examine quantitative face validity. Impact score had ranged from 1.2 to 5. Therefore, the pre-final version of the questionnaire containing 28 items was preserved for the next steps of psychometric assessment. In the qualitative face validity, participants declared all items are understandable and they have no problem reading them.

#### Construct validity

Exploratory factor analysis (EFA) was used to assess construct validity. The Kaiser-Meyer-Olkin (KMO) and Bartlett’s test illustrated that the data was proper for factor analysis (KMO index =0.51, χ^2^=3835.938, *P*<0.001). Principal component analysis with varimax rotation explored five factors with Eigenvalues greater than 1 and factor loading equal to or greater than 0.3; accounting for 64% of the variance observed. The factor-loading matrix for patterns identified in the SEQIDW is shown in Table 3.

**Table 3:** Exploratory factor analysis of the SEQ-IDW

**Domains**	**Items**	**Item Number**	**Factor 1**	**Factor 2**	**Factor 3**	**Factor 4**	**Factor 5**	ICC*	**Cronbach’s alpha coefficient**
	In public opinion, a divorcee is trouble for her neighbors.	6	0.80	0.26	0.06	0.07	0.07	0.10	
Discriminative marital status	To fulfill her future expenditures (rent, children’s education & marriage) a divorcee is in need of financial support.	11	0.89	0.03	0.15	0.07	−0.027	0.98	
	Divorced women are not given equal employment opportunities.	12	0.84	0.15	0.13	0.06	−0.16	1.00	
	Being insured is a kind of social support for divorcees.	13	0.90	0.18	0.12	0.11	0.01	0.10	
	Our society does not support divorcees.	14	0.59	0.22	0.09	0.16	0.01	0.99	
	When it comes to a divorcee, social justice is not adhered to.	15	0.85	0.01	0.07	0.13	0.01	0.10	
	A divorcee does not enjoy her family’s financial support.	18	0.58	0.36	0.07	0.21	0.25	0.10	
	Cancellation of a rental agreement is considered a sort of social exclusion for a divorcee.	20	0.33	0.15	0.04	0.43	0.00	1.00	
Economic dependence on marital status	Divorcees are abused at their work-places.	1	0.00	.57	.05	.37	0.48	1.00	0.79
	The society eyes divorcees in a disagreeable manner.	4	0.21	.76	.08	.28	0.17	1.00	
	Divorce carries stigma.	5	0.11	0.54	0.17	0.04	0.14	1.00	
	A divorcee feels like a burden.	7	0.54	0.41	.03	0.22	0.16	0.99	
	Divorced women are not treated the same as married women at the work-place.	8	0.1	0.70	0.15	0.19	0.20	1.00	
	Being a divorcee is a barrier to equal employment opportunities.	9	0.20	0.83	0.17	0.01	0.05	0.99	
	Unemployment and financial difficulties are amongst the most significant problems divorcees face.	10	0.25	0.77	0.01	0.02	0.09	0.96	
Exclusionary marital status	A divorcee is rejected by her family.	17	0.61	0.08	0.09	0.04	0.34	0.98	0.80
	A divorcee is deprecated by her family and children. =21	21	0.21	0.09	0.48	0.17	0.31	1.00	
	A woman’s divorced status is problematic for her child’s marriage.	22	0.19	0.09	0.89	0.06	0.01	1.00	
	A divorcee is more prone to risky behavior such as addiction and prostitution.	23	0.09	0.00	0.86	0.18	0.04	0.99	
	A divorcee’s financial needs force her to give in to open sexual relationships and/or to sell her body organs.	24	0.14	0.02	0.88	0.10	0.10	1.00	
	A divorcee is forced to do difficult and low-paying jobs to fulfill her financial needs. =25	25	0.11	0.13	0.36	0.17	0.70	1.00	
	A divorcee has a lower social rank, hence earning less income.	26	0.07	−0.00	0.67	0.19	0.16	1.00	
Traumatic marital status health risks	Our society does not welcome divorcees.	2	−0.04	0.36	0.11	0.70	0.29	1.00	0.70
	Our society does not understand divorcees.	3	0.01	0.02	0.19	0.68	0.22	1.00	
	Divorce is not an accepted phenomenon among families.	16	0.33	0.05	−0.06	0.62	0.001	0.97	
	Friends, family, and relatives avoid a divorcee.	19	0.46	0.18	0.09	0.67	0.03	0.98	
Frightening marital status	Married women avoid divorced women.	27	0.18	0.04	0.10	0.40	0.57	0.99	0.69
	A divorcee avoids those surrounding her.	28	0.01	0.07	0.84	0.09	0.92	0.99	

* 1: Intra-class correlation coefficient

Of 47 initial items, 19 items were omitted due to inappropriate factor loading and 28 items were remained. Extracted factors were as follow: Factor 1 (Discriminative marital status) with 8 items, including item 6, 11, 12, 13, 14, 15, 18, and 20.

Factor 2 (Economic dependence on marital status) with seven items, including item 1, 4, 5, 7, 8, 9, and 10.
Factor 3 (Exclusionary marital status). with 7 items, including item 17, 21, 22, 23, 24, 25, and 26.
Factor 4 (Traumatic marital status health risks) with 4 items, including item 2, 3, 16, and 19.
Factor 5 (Frightening marital status) with 2 items, including item 27 and 28 (Table 3).

### Reliability

Internal consistency was applied to assess reliability. The Cronbach’s alpha coefficient for the questionnaire was 0.87 and for the subscales, ranging between 0.70 and 0.88 cut off point. Furthermore, the ICC for the questionnaire was found to be 0.85 (good to excellent), providing the stability of the questionnaire.

## Discussion

The purpose of this study was to develop psychometric properties of SEQ-IDW. SEQ-IDW indicates that several factors must be considered while constructing a questionnaire in divorce assessment as a complex phenomenon. Despite economic consequences, divorce will affect women psychologically, socially, and physically (9, 24–26). The questionnaire was valid and reliable. The CVI and the CVR displayed reasonable content validity. In addition, the Cronbach’s alpha coefficient and ICC were acceptable and indicated good reliability and stability for the questionnaire. The final 28-item SEQ-IDW contained five subscales including discriminative marital status, Economic dependence on marital status, exclusionary marital status, Traumatic marital status (health risk), and frightening marital status. Items included in ‘Discriminative marital status’ subscales reflects inequality which occurs after a marital status change by separation such as job opportunities problems for a divorced woman. ‘Economic dependence on marital status’ subscale levels on insecure financial situations which emerge after separation for woman dramatically. In fact, the financial dependency of a woman will be threatened after divorce. Items in the ‘Exclusionary marital status’ subscale referred to social rejection particularly. In comparison to a married woman, a divorced woman is socially undesirable. Items included in ‘Traumatic marital status (health risks)’ shows probable health risk behavior, which jeopardizes woman health after divorce. Some woman after divorce is obliged to earn money through temporary marriage and sex working. The ‘Frightening marital status’ subscale displays items on the concept of divorce culturally. In another word, a divorced woman is a threat to a married woman and married women avoid divorced women.

There are some questionnaires to assess the women’s post-divorce life. Fisher Divorce Adjustment Scale (FDAS) is a psychological diagnostic questionnaire contains 100 items aimed to identify the strengths and weaknesses of the post-divorce adaptation of divorcees (27). The scale included seven subscales; adjustment to ending of a love relationship, feeling of self-worth, disentanglement from love relationship, feeling of self-anger, symptoms of grief, rebuilding social trust, and social self-worth. In compared to FDAS with a psychological perspective post-divorce life, our questionnaire, SEQ-IDW, focused on social aspects of divorce. Moreover, SEQ-IDW is a gender specific questionnaire comparing FDAS.

Another tool, Co-Parenting Behavior Questionnaire (CBQ) aimed to assess co-parenting interactions of divorced parents from their child’s perspective (28). The CBQ contains 86 items with reflecting parental interaction variables, father-parenting variables, and mothers-parenting variables CBQ like as FDAS can be psychologically assess post-divorce life while SEQ-IDW reflects divorced woman’s viewpoint socially. Furthermore, SEQ-IDW is a self-reported tool for women especially but CBQ is a questionnaire to assess the post-divorce consequences from child’s perspective.

There is not questionnaire particularly to address women’s social exclusion but some tools such as Discrimination and Stigma Scale (DISC) can be assessed discrimination and stigma as variables related to the concept of exclusion. DISC was developed in patients with mental illness including 35 items in 4 subscales as unfair treatment, stopping self, overcoming stigma, and positive treatment (29). Although DISC can be assessing the exclusion implicitly, the main scope of this questionnaire is discrimination and stigmatizing. Comparing with DISC, exclusion is explicitly assessed by SEQ-IDW. The current study had limitations, as we did not test the convergent or known group’s validity of SEQ-IDW.

The results of the present study may be useful in for women health promoting programs development and a suitable scale for assessment of divorced women health in social dimension. Further studies in various populations of women are needed to establish stronger psychometric properties for the questionnaire. In addition, more assessment especially confirmatory factor analysis is suggested.

## Conclusion

This study provided preliminary evidence for the psychometric properties of the SEQ-IDW, so it could be applicable to assess the of the women’s social exclusion post-divorce life.

## Ethical considerations

Ethical issues (Including plagiarism, Informed Consent, misconduct, data fabrication and/or falsification, double publication and/or submission, redundancy, etc.) have been completely observed by the authors.
